# 

**Published:** 1904-09-24

**Authors:** 


### NOTICE TO CORRESPONDENTS.

All MSS., Letters, Books for Review, and other matters intended for the Editor should be addressed The Editor, "The Hospital," The Hospital Buildings, 28 & 29 Southampton Street, Strand, W.C.

The Editor cannot undertake to return rejected MS., even when accompanied by stamped directed envelope.

**Notice to Advertisers and Subscribers.**

All Advertisements, Orders for Copies of The Hospital, and Business Communications should be addressed to **THE MANAGER (Not to the Editor),** The Hospital, 28 & 29 Southampton Street, Strand, London, W.C.

The Cost of Subscription, post free, is as follows:—Per week, 3½d.; per quarter, 3s. 3d.; per half-year, 6s. 6d.; per annum, 13s. Foreign and Colonial:—Per annum, 19s. 6d., payable, in all cases, in advance.

**NOTICE.—Vol. XXXV. of "The Hospital" (October 1903 to March 1904), in handsome cloth binding, is now on sale at the office of this Journal, price 10s. 6d. Cases for binding the Numbers contained in this Volume 2s. Index to Vol. XXXV., 3½d., post free.**

**The Monthly part for October will be ready on Oct. 1. Price 1s., by post, 1s. 3d.**

### Medical Appointments.

E. S. Bell, M.D.Brux., M.R.C.S., L.R.C.P., Medical Superintendent, Bermondsey Parish Infirmary. G. W. Bampfylde Daniell, M.R.C.S., L.R.C.P.Lond., Anæsthetist to the Edinburgh Dental Hospital. Harold A. Haig, M.R.C.S., L.R.C.P.Lond., Junior Assistant Medical Officer to the Cumberland County Council. D. Hethcote, M.B., B.S.Durh., District Medical Officer, Richmond (Yorks) Union. A. H. Holmes, M.D., District Medical Officer of the Bourne Union. John Hoole, M.R.C.S., L.S.A., Medical Officer of Health to the Ashbourne Rural District. J. K. Howlett, L.R.C.P.&S.Edin., L F.P.S.Glasg., Medical Officer and Public Vaccinator for the Grassenhall District of the Mitford and Launditch Union. B. M. Hughes, M.R.C.S., L.R C P.Lond., District Medical Officer of the Forehoe Incorporation. F. W. Jackson, M.R.C.S., L.R.C.P.Lond., Certifying Factory Surgeon for the Market Rasen District, Lincolnshire. W. Kemp, M.B., C.M.Edin., Certifying Factory Surgeon for the Castleford District, County York. J. W. Killen, M.B., B.S., R.U.I., Assistant Medical Officer at the Hendon Asylum of the Central London Sick Asylum District. W. W. J. Lawson, M.B., Ch.B.Aberd., Second Assistant Medical Officer to the Berkshire County Asylum, Wallingford. G. K. Levick, M.B., B.S.Lond., Medical Officer of Health to the Havant District Council. James E. F. MacDonald, M.B., B.S.Melb., Acting Assistant Health Officer for the Port of Brisbane. T. T. Macklin, M.D.Glasg., Certifying Factory Surgeon for the Whalley District, Counter Lancaster, and Medical Officer of Health, Clitheroe Rural District. John N. E. MacLennan, M.B., M S.Aberd., Government Medical Officer and Vaccinator at Young, N.S.W. H. Markby, M.R.C.S.Eng., L R.C.P.Lond., Medical Officer to the Post Office, Morley District. R J. Mills, M.B., C.M.Aberd., Medical Officer for the Thorpe District of the Blofield Union. T. H. Molesworth, M.B., B.C.Camb., District Medical Officer of the Dover Union. D. Moodie, L.R.C P. & S.Edin., District Medical Officer of the Sedgefield Union. E. J. F. Moore, M.R.C.S., L.R.C.P., Medical Officer, Well Street Temporary Workhouse of the West Ham Union. Sydney G. Mostyn, M.B., B.Ch.-Oxon., D.P.H Camb., Medical Officer of Health for the Borough of South Shields. F. D. Nicholson, B.C.Camb., District Medical Officer of the Hoxne Union. A. S. Norman, L R.C.P., M.R.C.S.-Eng., D.P.H.Camb., Certifying Factory Surgeon for the Havant District, Southampton. L. E. Parkhurst, M.D., B.Ch.Oxon., Medical Officer of the Workhouse of the Brackley Union. E. A. Perram, M.D.Lond., M.R.C.S., Certifying Factory Surgeon for the Whitchurch District, County Salop. L. Potts, M.R.C.S.Eng., Medical Officer for the Headley District of the Epsom Union. F. A. Rodway, M.B., B.S.Melb., Junior House Surgeon to the Hobart Hospital, Tasmania. A. P. Ross, M.B., M.S.Edin., Government Medical Officer and Vaccinator at Condobolin, N.S.W. D. M. Ross, L.R.C.P.Lond., Assistant Medical Officer, St. Marylebone Infirmary. Gordon Ross, M.B., B.S.Melb., Resident Medical Officer at the Brisbane Hospital, Queensland. Thomas Sayer, M.R.C.S.Eng., L R.C.P.Lond., Medical Officer and Public Vaccinator for the Kirklington District of the Bedale Union and for the Pickhill District of the Thirsk Union; also Medical Officer of Health for Kirklington, Yorks. David Shannon, M.B., Ch.B.Glasg., L.M.(Rot. Dub.), Indoor House Surgeon to the Maternity Hospital, Glasgow. Edward S. Sharpe, M.B, Ch.B.Edin., Junior Assistant House Surgeon to the Stockport Infirmary. G. M. Soper, M.R C.S., L.R.C.P., District Medical Officer of the Totnes Union. J. H. Stacy, L.R.C.P.&S.Edin., Medical Officer of the Receiving Home of the Norwich Union. P. R. Tarbet, M.R.C.S., L.R C.P.Lond, District Medical Officer of the Hitchin Union. G. H. Wickham, M.B., B C.Camb, Certifying Factory Surgeon for the Fleet District, County Southampton. J. O. Williams, M.R.C.S., L.R.C.P., Medical Officer of Health for Barmouth Urban District.

### "The Hospital" Institutional Library.

**"Hospitals and Asylums of the World." 4 Vols., with a Portfolio of Plans, complete £12 12s.**

**"Burdett's Hospitals and Charities, 1904." 5s. net.**

**"Burdett's Official Nursing Directory." 8s. net.**

**"Cottage Hospitals, General, Fever, and Convalescent." 10s. 6d.**

**"Uniform System of Accounts for Hospitals and Public Institutions." New Edition. 4s. net.**

**"Hospital Expenditure: The Commissariat." 2s. 6d.**

**"A Legal Handbook for the Use of Hospital Authorities." 2s. 6d.**

**"The Cottage Hospital Case Book or Register of Patients." 10s. and 12s. 6d.**

**All these are published by the Scientific Press, Ltd., and may be obtained through any bookseller, or direct from the publishers, 28 and 29 Southampton Street, Strand, London, W.C.**

### IMPORTANT NURSING TEXT-BOOKS +

**A HANDBOOK FOR NURSES.**
By J. K. Watson, M.D., M.B., C.M.
New and Revised Edition.
**5s.** net, **5s. 4d.** post free.

**NURSING: Its Theory and Practice.**
Being a complete Text-book of Medical, Surgical, and Monthly Nursing.
By Percy G. Lewis, M.D., M.R.C.S., L.S.A.
**3s. 6d.** post free.

**SURGICAL WARD-WORK AND NURSING.**
By Alexander Miles, M.D.Edin., C.M., F.R.C.S E.
Copiously illustrated.
**3s. 6d.** net, **3s. 10d.** post free.

**OPHTHALMIC NURSING.**
By Sydney Stephenson, M.B., F.R.C.S.E.
With a list of Instruments required, and a Glossary of Terms.
**3s. 6d.** net, **3s. 10d.** post free.

**ELEMENTARY ANATOMY AND SURGERY FOR NURSES.**
By William McAdam Eccles, M.S.Lond., M.B., F.R.C.S., L.R.C.P.
Profusely illustrated.
**2s. 6d.** post free.

**ELEMENTARY PHYSIOLOGY FOR NURSES.**
By C. F. Marshall, M.D., B.Sc., F.R.C.S.
Illustrated.
**2s.** post free.

**NURSING IN DISEASES OF THE THROAT, NOSE, AND EAR.**
By P. MacLeod Yearsley, F.R.C.S.
Illustrated.
**2s. 6d.** post free.

**HOSPITAL SISTERS AND THEIR DUTIES.**
By Eva C. E. Lückes, Matron, London Hospital.
**2s. 6d.** post free.

**OUTLINES OF INSANITY.**
By Francis H. Walmsley, M.D.
**1s. 6d.** post free.

The Latest and Best Book on Midwifery.
**A COMPLETE HANDBOOK OF MIDWIFERY.**
By J. K. Watson, M.D.Edin., Author of "A Handbook for Nurses."
With 143 Illustrations.
**6s.** net, **6s. 4d.** post free.

**A PRACTICAL HANDBOOK OF MIDWIFERY.**
By Francis W. Haultain, M.D.
Profusely illustrated. Handsomely bound in leather. **6/-** net, **6,3** post free.

**PRACTICAL GUIDE TO SURGICAL BANDAGING AND DRESSINGS.**
By Wm. Johnson Smith, F.R.C.S.
Profusely illustrated (suitable for apron pocket), cloth. **2s.** post free.

**THE EXAMINATION OF THE URINE.**
By J. K. Watson, M.D., M.B., C M., Author of "A Handbook for Nurses."
**1s.** net, **1s. 1d.** post free.

**ART OF FEEDING THE INVALID.**
By a Medical Practitioner and a Lady Professor of Cookery.
**1/6** post free.

**THE NURSE'S PRONOUNCING DICTIONARY OF MEDICAL TERMS AND NURSING TREATMENT.**
Compiled for the Use of Nurses.
By Honnor Morten.
Fifth Edition, with phonetic pronunciation, Edited and Revised by Mary I. Burdett.
(Suitable for apron pocket).
**2/-** post free.

**THE HUMAN BODY.**
Its Personal Hygiene and Practical Physiology.
By B. P. Colton, Professor of Natural Science, Illinois University.
Profusely illustrated. **5s.** post free.

**ART OF MASSAGE.**
By Mrs. Creighton Hale.
**6s.** post free.

**MEDICAL GYMNASTICS (including the Schott (NAUHEIM) Movements).**
Being a Text-book of Massage and Mechanical Therapeutics Generally.
By Axel V. Grafstrom, B.Sc., M.D.
Illustrated. **2s. 6d.** post free.

NEW CATALOGUE OF NURSING MANUALS POST FREE ON APPLICATION.

*The above Works can be obtained of all Booksellers, or of*

